# Pulmonary Infection Associated with *Mycobacterium canariasense* in Suspected Tuberculosis Patient, Iran

**DOI:** 10.3201/eid2510.190156

**Published:** 2019-10

**Authors:** Fatemeh Sakhaee, Farzam Vaziri, Golnaz Bahramali, Kambiz Taremian, Seyed Davar Siadat, Abolfazl Fateh

**Affiliations:** Pasteur Institute of Iran, Tehran, Iran (F. Sakhaee, F. Vaziri, G. Bahramali, S.D. Siadat, A. Fateh);; Mohammad Medical Imaging Center, Varamin, Iran (K. Taremian)

**Keywords:** Antimicrobial resistance, bacteria, Iran, multilocus sequence analysis, *Mycobacterium canariasense*, pulmonary infection, respiratory infections, tuberculosis, tuberculosis and other mycobacteria

## Abstract

*Mycobacterium canariasense* had only been isolated in humans from blood and contaminated catheters. We report a case of pulmonary disease associated with *M. canariasense* infection that was identified by multilocus sequence analysis; the illness was initially ascribed to *M. tuberculosis*. *M. canariasense* should be considered a cause of respiratory infection.

*Mycobacterium tuberculosis* is a widely known cause of pulmonary disease, specifically tuberculosis (TB). However, its symptoms may be similar to those of pulmonary infections caused by other pathogens. We document a case in which disease initially ascribed to *M. tuberculosis* was ruled out through testing and a different mycobacterium, *M. canariasense*, was identified as the likely cause. 

The patient was a 67-year-old woman with pulmonary infection living in a village in Afghanistan who traveled to Iran for treatment. Her signs and symptoms included fever, cough, sputum, weight loss, chest pain, and night sweats; the fever and cough had persisted for 6 months. Forty-six years earlier, at 21 years of age, she had experienced a pulmonary TB episode, which was treated with anti-TB drugs. She had no history of smoking or taking immunosuppressive drugs. No other pulmonary diseases were reported. 

Results of clinical parameters were normal, apart from an elevated C-reactive protein (CRP) rate (72.4 mg/L) and erythrocyte sedimentation (ESR) rate (85 mm/h). The induration from a tuberculin skin test was 21 mm. A computed tomography scan indicated calcified mediastinal lymph nodes, nodular opacities, and fibrotic changes in the left lower lobe (Appendix Figure). The physician assumed a TB reactivation, and chemotherapy was initiated with isoniazid, rifampin, ethambutol, and pyrazinamide. However, the pulmonary symptoms did not disappear after 4 months. 

Five sputum samples from the patient were sent to the Pasteur Institute of Iran (Tehran, Iran) in August 2017 for *M. tuberculosis* testing. Results of smear tests indicated partially acid-fast bacilli, whereas all of the samples showed negative results when evaluated by insertion sequence 6110 PCR assay for *M. tuberculosis*. A culture of sputum samples on Lowenstein-Jensen medium after 3 days showed rapidly growing mycobacteria with smooth, small, shiny, and nonpigmented colonies, which turned pale yellow and shinier after 4 days. Phenotypic tests of the isolate were positive on MacConkey agar without crystal violet, urease, Tween 80 hydrolysis, heat-resistant catalase, and arylsulfatase tests. On the other hand, nitrate reductase, growth in 5% NaCl, tellurite, and niacin accumulation tests were negative. 

Multilocus sequence analysis was performed for partial *hsp65* and *rpoB* genes and full *16S rRNA* gene, as described in the literature (*1*–*3*), and results indicated 100% homology to *M. canariasense* (Figure). This organism was described by Jiménez et al. in a suspected nosocomial outbreak infecting 17 patients during January 2000–September 2002 at a tertiary care hospital in the Canary Islands in Spain (*4**,**5*). The phenotypic and genotypic characteristics of the isolate agreed with those for *M. canariasense*, on the basis of guidelines of the American Thoracic Society and the Infectious Disease Society of America (*6*).

**Figure:**
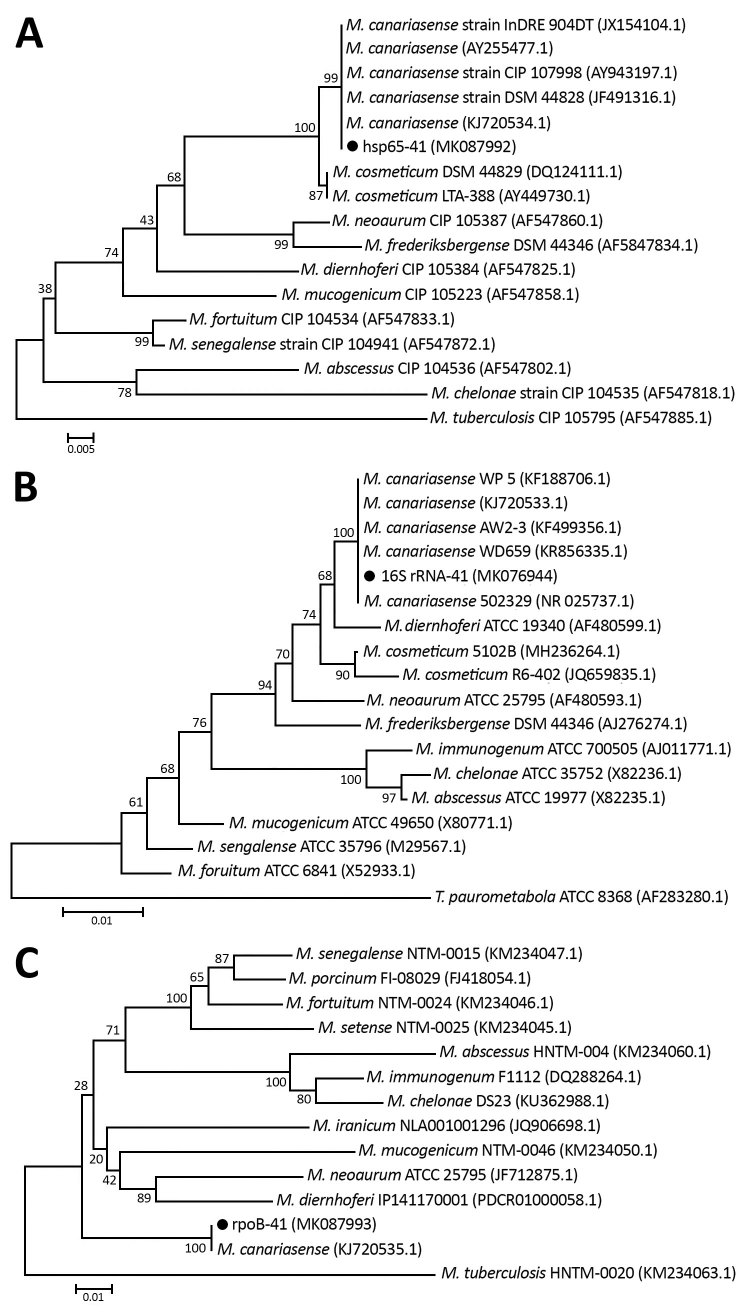
Neighbor-joining tree of the *hsp65* (A), *16S rRNA* (B), and *rpoB* (C) genes of an isolate from a patient infected with *Mycobacterium canariasense*, Tehran, Iran (black dots), and other rapidly growing mycobacteria. Outgroup for *hsp65*/*rpoB* genes was *Mycobacterium tuberculosis* and for the *16S* rRNA gene was *Tsukamurella paurometabola*. Bootstrap values are represented on branch nodes. GenBank accession numbers are given in parentheses for reference sequences. The nucleotide sequences identified in this study were submitted to GenBank under the following accession numbers: *hsp65*, MK087992; *rpoB*, MK087993; and *16S* rRNA, MK076944. Scale bars indicate nucleotide substitutions per site.

The results of susceptibility testing, performed according to Clinical and Laboratory Standards Institute guidelines (*7*), indicated that the *M. canariasense* isolate was highly resistant to isoniazid, rifampin, ethambutol, and streptomycin; extremely susceptible to amikacin, levofloxacin, clarithromycin, cefoxitin, ciprofloxacin, imipenem, doxycycline, minocycline, and trimethoprim/sulfamethoxazole; and intermediately susceptible to vancomycin. On the basis of these results, the patient was treated with levofloxacin and amikacin for 17 days. After treatment, sputum samples were collected from the patient over 5 days. The results of smear and culture tests were negative for partially acid-fast bacilli, and the results of a computed tomography scan and CRP and ESR measurements were normal.

Previously, *M. canariasense* had only been detected in the blood of patients and in contaminated catheters (*8*,*9*); no study had reported pulmonary infections associated with this isolate. Our results reveal that clinical and radiographic findings of *M. canariasense* pulmonary infection are similar in appearance to those of TB and other nontuberculous mycobacteria infections. However, these findings and improved radiological findings and ESR and CRP levels due to chemotherapy, indicating that *M. canariasense* had been eliminated, strongly suggest that the bacterium could have been the cause of pulmonary disease in this patient. 

Although no specific treatment has been recommended for *M. canariasense* infection, combination therapy with levofloxacin and amikacin produced a successful outcome in this case; no recurrent pulmonary disease was reported in the patient. However, treatment with other drugs to which *M. canariasense* is susceptible might also succeed. In a 2006 report, Campos-Herrero et al. noted the favorable outcomes produced by fluoroquinolones and amikacin (*8*). However, the optimal antimycobacterial regimen for *M. canariasense* infection needs to be clearly established in more cases. 

Appendix Additional information related to pulmonary infection associated with *Mycobacterium canariasense* in a suspected tuberculosis patient, Iran.
