# Supplementary material for: Pulmonary Infection Associated with Mycobacterium canariasense in Suspected Tuberculosis Patient, Iran
Source: Emerg Infect Dis. 2019 Oct;25(10):1984–6. doi: 10.3201/eid2510.190156 (PMC6759235; doi:10.3201/eid2510.190156)
Supplement: Appendix — Additional information related to pulmonary infection associated with Mycobacterium canariasense in a suspected tuberculosis patient, Iran. [file 19-0156-Techapp-s1.pdf]

Article DOI: https://doi.org/10.3201/eid2510.190156

Pulmonary Infection Associated with *Mycobacterium canariasense* in Suspected Tuberculosis Patient, Iran

Appendix

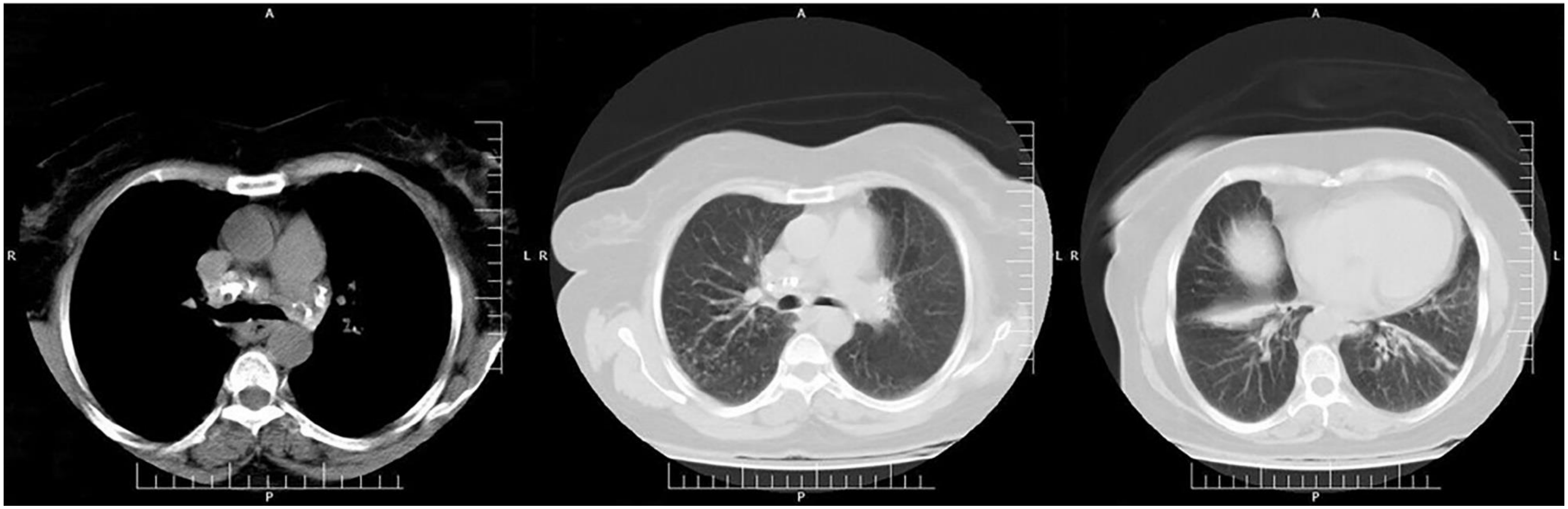

Figure: Chest CT scan image indicating calcified mediastinal lymph nodes, nodular opacities on both sides, a linear band in both lower lobes, partial wedge-shaped collapse in the right lower lobe, and fibrotic changes in the left lower lobe.
